# Ultrasonic evaluation of renal cortex arterial area enables differentiation between hypertensive and glomerulonephritis-related chronic kidney disease

**DOI:** 10.1007/s11255-017-1634-7

**Published:** 2017-06-01

**Authors:** Arkadiusz Lubas, Grzegorz Kade, Robert Ryczek, Piotr Banasiak, Przemysław Dyrla, Katarzyna Szamotulska, Daniel Schneditz, Stanisław Niemczyk

**Affiliations:** 1 Department of Internal Diseases, Nephrology and Dialysis, Military Institute of Medicine, Szaserów 128, 04-141 Warsaw 44, Poland; 2 Department of Cardiology, Military Institute of Medicine, Szaserów 128, 04-141 Warsaw 44, Poland; 3 Health Center Karczew, Otwocka 28, 05-480 Karczew, Poland; 4 Department of Gastroenterology, Military Institute of Medicine, Szaserów 128, 04-141 Warsaw 44, Poland; 5 Department of Epidemiology, Institute of Mother and Child, Kasprzaka 17a, 01-211 Warsaw, Poland; 6 Institute of Physiology, Medical University of Graz, Harrachgasse 21/5, 8010 Graz, Austria

**Keywords:** Renal perfusion, Hypertension, Arterial rarefaction, Ultrasound Doppler

## Abstract

**Purpose:**

Identifying the primary etiology of cardio-renal syndrome in a timely manner remains an ongoing challenge in nephrology. We hypothesized that hypertensive kidney damage can be distinguished from chronic glomerulonephritis at an early stage of chronic kidney disease (CKD) using ultrasound (US) Doppler sonography.

**Methods:**

Fifty-six males (age 54 ± 15, BMI 28.3 ± 3.5 kg/m^2^) with hypertension and stable CKD at stages 2–4 [38 with essential hypertension (HT-CKD); 18 with glomerulonephritis (GN-CKD)] were studied. Blood tests, UACR, echocardiography, ABPM, carotid IMT, and an ultrasound dynamic tissue perfusion measurement (DTPM) of the renal cortex were performed.

**Results:**

HT-CKD patients had reduced proximal renal cortex perfusion as well as reduced total and proximal renal cortex arterial area. Proximal renal cortex arterial area ≤0.149 cm^2^ identified hypertension-related CKD with a sensitivity of 71% and a specificity of 78% (AUC 0.753, *p* < 0.001).

**Conclusions:**

Evidence of diminished arterial vascularity or perfusion of renal proximal cortex, both derived from US Doppler, could be helpful in differentiating hypertensive nephropathy from glomerulonephritis-related CKD.

## Introduction

Changes in renal perfusion play an important part in the development of cardio-renal syndrome [1, 2]. Both long-standing hypertension and a history of malignant hypertension are well known to induce chronic kidney damage. On the other hand, kidney disease may cause or aggravate an existing hypertension. Vascular changes in the course of hypertension are significant diagnostically and constitute important additional cardiovascular risk factors [3]. In large arteries such changes include thickening of the whole vessel wall and its intima-media layers. In small arteries changes are characterized by wall hypertrophy and vascular rarefaction [4]. It also has been shown that cardiac dynamics have a significant influence on renal perfusion and function [5, 6]. On the other hand, etiology of renal impairment and related secondary hypertension due to chronic, non-vascular-mediated glomerulonephritis is different.

Within the last years there has been significant progress in the field of ultrasound (US) imaging and the widespread availability of advanced US equipment has considerably extended the potential of US Doppler diagnostics. It has been recently shown that dynamic Doppler perfusion measurements (DTPM) can be used to differentiate between inflammatory and proliferative changes in the pancreas [7]. US-derived markers of renal perfusion disorders have been used to diagnose focal lesions such as abscesses, cysts, and tumors, as well as lesions affecting the whole kidney such as ischemic and diabetic nephropathy, transplant rejection, and obstructive uropathy, or cardiac disorders such as hypertensive organ damage or systolic heart failure [8–11].

We therefore hypothesized that vascular-induced hypertensive kidney damage leads to perfusion changes that can be distinguished from those of chronic glomerulonephritis. The aim of the study was to evaluate the differences in US Doppler parameters of renal cortex perfusion in hypertensive and glomerulonephritis-related kidney damage.

## Materials and methods

Patients with hypertension and stable CKD during last 3 months, at stages 2–4 were recruited from consecutive subjects admitted to the Department of Internal Diseases Nephrology and Dialysis. Exclusion criteria were defined as (a) cause of kidney damage other than glomerulonephritis or hypertension, or (b) the presence of acute cardiac and renal diseases, or (c) history of renal artery stenosis, CKD stage 5, heart failure in NYHA III or IV stage, atrial fibrillation, hyperkinetic state, inflammation, connective tissue diseases, vasculitis, diabetes mellitus, amyloidosis, hydronephrosis, systemic cancer, or (d) inability to obtain good-quality US images of renal or cardiac structures. Stages of CKD were defined as per K/DOQI guidelines [12]. On the basis of anamnesis, previously and actually performed blood and urinary tests patients were classified into hypertension-related kidney damage group (HT-CKD) if they had long-standing or history of severe hypertension preceding the renal impairment without nephrotic proteinuria. Patients with previously recognized glomerulonephritis (performed renal biopsy or CKD with persistent microhematuria and/or proteinuria and excluded other causes of kidney damage) were classified into GN-CKD group.

The local bioethics committee approved the protocol of the study (35/WIM/2011, June 15, 2016). All participants enrolled in the study provided written informed consent.

### Blood and urinary tests

Serum creatinine (Cr) and cystatin C (Cys) were measured, and glomerular filtration rate was estimated (eGFR) using the chronic kidney disease epidemiology (CKD-EPI) formula [13]. The presence of inflammation and cardiac function was assessed by measuring C-reactive protein (CRP), Troponin-I, and N-terminal prohormone of brain natriuretic peptide (NT-proBNP) levels. Urinary albumin-to-creatinine ratio (mg/dL/mg/dL) (UACR) from first-morning urine sample was calculated.

### Blood pressure monitoring

Systolic and diastolic blood pressures were measured every 15 min at daytime and every 30 min at night using the ambulatory blood pressure monitor (ABPM-04, Meditech, Hungary) and averaged over the whole daily cycle.

### Kidney ultrasound

A Logiq P6 (GE Healthcare, Korea) US device and a convex transducer 4L (2–5 MHz) were used for a renal examination. Maximal right renal length and mean cortical thickness (2 measurements in upper and lower pole) in longitudinal axis were examined. Renal resistance indices (RI) in segmental arteries of both kidneys were calculated from two to three flow measurements in the upper, middle, and lower regions of the renal sinus. When the clinical data suggested renal artery stenosis, a full Doppler examination of renal and intrarenal arteries was performed.

Color Doppler renal cortical perfusion assessments were made as described elsewhere [5, 6]. Longitudinal sections of right kidneys were examined because of improved access to the mid-segment of the renal cortex. Left kidneys were not examined to avoid possible artifacts caused by preceding renal biopsies. Renal perfusion was estimated using the DTPM method [14]. Renal arterial perfusion (mL/s) and renal arterial area (cm^2^) obtained from the best quality color Doppler video clip, selected from 2 to 3 recorded DICOM sequences, covering a minimum of three heart cycles in the chosen region of interest (ROI) were calculated using the PixelFlux (Chameleon-Software, Leipzig, Germany) software package. Color Doppler frequency was constantly set at 3.4 MHz. The ROI was set in the mid-segment of the renal cortex between the outer border of medullary pyramids and the kidney surface avoiding focal abnormalities. The ROI sample contained vessels pointing to the transducer so that angle correction was not required. Total renal cortex perfusion (RCP) and total renal cortex arterial area (RCAA) were calculated from the whole ROI. Then the ROI was divided into equally sized proximal and distal regions. Regional perfusions and arterial areas were separately measured and then considered for statistics. To avoid errors resulting from different ROI sizes, proximal renal cortex arterial area was normalized to proximal ROI creating an arterial area index (AAI). This index is thought as a measure of arterial vascularity, a substitute of 3D arterial density in 2D US.

### Carotid sonography

To determine the degree of hypertensive organ damage left common carotid artery intima-media thickness (cIMT) was measured manually at least 10 mm upstream of the carotid sinus using a 11L transducer (10–13 MHz) [5]. The mean of three measurements was calculated.

### Cardiac sonography

For echocardiography the Vivid S6 system (GE Healthcare, USA) was used with a M4S-RS transducer (1.5–3.6 MHz). All M-mode measurements were taken according to recommendations of the American Society of Echocardiography [15]. The left atrial (LA) diameter was measured using M-mode methodology from the parasternal long axis view. Left ventricular mass (LVM) was calculated from the Devereux et al. formula and then normalized (LVMI) for Mosteller’s body surface area [16, 17]. Left ventricular ejection fraction (LVEF) was estimated by Simpson’s biplane method [18]. Left ventricular cardiac index (CI) was collected using 2D/continuous US Doppler functions. Peak values of early (E) and late (A) parts of mitral inflow velocities were measured. Tissue Doppler early diastolic mitral annular velocity (E′) was measured from the apical four-chamber view with a 4-mm sample volume placed at the septal corner of mitral annulus. Then E/A and E/E′ ratios were calculated.

### Statistical analysis

Variables were analyzed with Pearson’s or Spearman’s correlation test depending on the type of distribution. Accordingly, the parametric *T* test or the nonparametric U Mann–Whitney test was used to analyze the difference of continuous variables between groups. The Chi-square test was performed to determine differences in hypertensive treatment. Stepwise multivariable linear regression analyses were used to identify the factors independently associated with proximal RCP and proximal RCAA. Receiver operating characteristic (ROC) analyses were performed to identify thresholds of proximal RCP and RCAA to differentiate between hypertensive and glomerulonephritis-related chronic kidney disease. Statistica 12 (StatSoft Inc., Poland) software was used for statistical analysis.

## Results

Sixty males with hypertension and stable CKD ranging from stages 2 to 4 entered the study. Four patients were excluded, three because of unexpected diabetes mellitus and one patient because of failure to obtain echocardiographic Doppler data, so that 56 patients (age 54 ± 15, BMI 28.3 ± 3.5 kg/m^2^) entered final analysis. Chronic kidney disease caused by essential hypertension (HT-CKD) was found in 38 patients, whereas chronic kidney disease originating from glomerulonephritis (GN-CKD) was found in 18 patients. 9 HT-CKD and 2 GN-CKD patients underwent full Doppler examinations of renal arteries with the exclusion of renal artery narrowing. In all patients the difference between right and left renal RI did not exceed 0,05 [19].

HT-CKD patients were older and had lower UACR, but the kidney length, cortical thickness, and function were similar in both groups (Table 1). Groups did not differ in antihypertensive or nephroprotective treatment (Table 2).

**Table 1:** Patient and renal characteristics

Data	Total (*n* = 56)	HT-CKD (*n* = 38)	HT-CKD matched (*n* = 18)	GN-CKD (*n* = 18)	*p* value HT/GN (HTm/GN)
Age (y)	53.96 ± 14.89	58.08 ± 13.37	47.4 ± 12.12	45.3 ± 14.11	0.003 (0.728)
BMI (kg/m^2^)	28.31 ± 3.46	28.83 ± 3.41	29.28 ± 3.95	27.26 ± 3.32	0.106 (0.114)
Cystatin (mg/L)	1.54 ± 0.72	1.42 ± 0.66	1.43 ± 0.62	1.73 ± 0.83	0.187 (0.351)
Creatinine (mg/dL)	1.83 ± 0.78	1.68 ± 0.73	1.78 ± 0.91	2.08 ± 0.81	0.054 (0.217)
CKD-EPICys-Cr (mL/min/1.73 m^2^)	54.29 ± 27.91	58.55 ± 28.79	63.00 ± 34.43	48.50 ± 25.90	0.210 (0.210)
Urea/creatinine (mg/dL/mg/dL)	36.36 ± 9.26	37.75 ± 9.96	34.28 ± 8.03	33.14 ± 6.22	0.188 (0.914)
UACR (mg/dL/mg/dL)	0.070 [0.000–2.134]	0.021 [0.000–0.665]	0.029 [0.004–0.665]	0.508 [0.003–2.134]	<0.001 (0.001)
CRP (mg/dL)*	0.19 [0.02–12.80]	0.21 [0.02–12.80]	0.21 [0.02–2.94]	0.14 [0.02–1.50]	0.140 (0.255)
Renal length (mm)	110.2 ± 10.5	110.5 ± 10.5	110.8 ± 6.0	109.5 ± 10.8	0.757 (0.693)
Cortical thickness (mm)	14.3 ± 2.9	14.4 ± 2.9	16.0 ± 2.5	14.1 ± 3.2	0.689 (0.069)

*BMI* body mass index; CKD-EPI—based on cystatin (Cys) and creatinine (Cr) chronic kidney disease epidemiology formula; GN-CKD—CKD due to glomerulonephritis; HT-CKD*—*hypertensive nephropathy; HTm*—*HT-CKD matched; * median [range]

**Table 2:** Comparison of antihypertensive treatment between investigated groups

Kind of medication	HT-CKD (*n* = 38) (%)	GN-CKD (*n* = 18) (%)	*p* value (HT/GN)
ACE-I	45.45	26.67	0.217
ARB	24.24	40.00	0.266
BB	73.53	46.67	0.069
CCB	45.45	40.00	0.724
Diuretics	78.79	66.67	0.369
A1B	24.24	13.33	0.388
CN	6.06	13.33	0.398

*ACE-I* angiotensin-converting enzyme inhibitor, *ARB* angiotensin receptor blocker, *A1B* α1 adrenergic receptor blocker (doxazosin), *BB* b-blocker, *CCB* calcium channel blocker, *CN* centrally acting agent (clonidine, α-methyldopa), *GN-CKD* CKD due to glomerulonephritis, *HT-CKD* hypertensive nephropathy

Patients were not hypertensive according to ambulatory blood pressure measurements, and groups did not differ in 24-h blood pressure parameters, but LA, CI, E/E′, and cIMT were higher in the HT-CKD group (Table 3).

**Table 3:** Cardiovascular characteristics of investigated groups

Data	Total (*n* = 56)	HT-CKD (*n* = 38)	HT-CKD matched (*n* = 18)	GN-CKD (*n* = 18)	*p* value HT/GN (HTm/GN)
Troponin-I (ng/mL)*	0.021 [0.002–0.390]	0.023 [0.006–0.390]	0.016 [0.006–0.390]	0.018 [0.002–0.360]	0.435 (0.792)
NT-proBNP (pg/mL)*	91.55 [10.60–27,785.0]	104.15 [12.2–27,785.0]	42.27 [12.2–27,785.0]	78.85 [10.6–392.8]	0.229 (0.486)
cIMT (mm)	0.837 ± 0.206	0.889 ± 0.197	0.787 ± 0.199	0.712 ± 0.177	0.004 (0.317)
LA (cm)	3.88 ± 0.52	3.97 ± 0.50	4.01 ± 0.50	3.67 ± 0.51	0.044 (0.056)
LVMI (g/m^2^)	103.56 ± 32.64	104.82 ± 35.54	112.64 ± 45.38	100.83 ± 25.99	0.952 (0.728)
LVEF (%)	60.79 ± 9.31	61.46 ± 8.79	61.59 ± 6.76	59.38 ± 10.45	0.450 (0.530)
CI (L/min/m^2^)	4.13 ± 1.21	4.40 ± 1.20	3.88 ± 1.21	3.57 ± 1.08	0.017 (0.575)
E/A	1.06 ± 0.45	1.00 ± 0.38	1.14 ± 0.44	1.18 ± 0.57	0.240 (0.945)
E/E′	10.00 ± 2.80	10.38 ± 2.55	10.44 ± 3.11	9.09 ± 3.27	0.020 (0.089)
SBP (mmHg)	126.14 ± 15.61	125.59 ± 17.20	127.88 ± 16.48	127.33 ± 11.82	0.525 (0.597)
DBP (mmHg)	76.65 ± 11.43	75.59 ± 12.56	79.82 ± 12.54	78.94 ± 8.36	0.063 (0.498)

*CI* cardiac index, *E/A* ratio of transmitral early (E) to late (A) ventricular filling velocities, *cIMT* carotid intima-media thickness, *E/E′* ratio of transmitral early filling velocity to tissue Doppler early diastolic mitral annular velocity, *GN-CKD* CKD due to glomerulonephritis, *HT-CKD* hypertension-related nephropathy, *HTm* HT-CKD matched, *LA* left atrium diameter, *LVEF* left ventricular ejection fraction, *LVMI* left ventricular mass index, *SBP, DBP* systolic, diastolic blood pressure; * median [range]

**Fig. 1:**
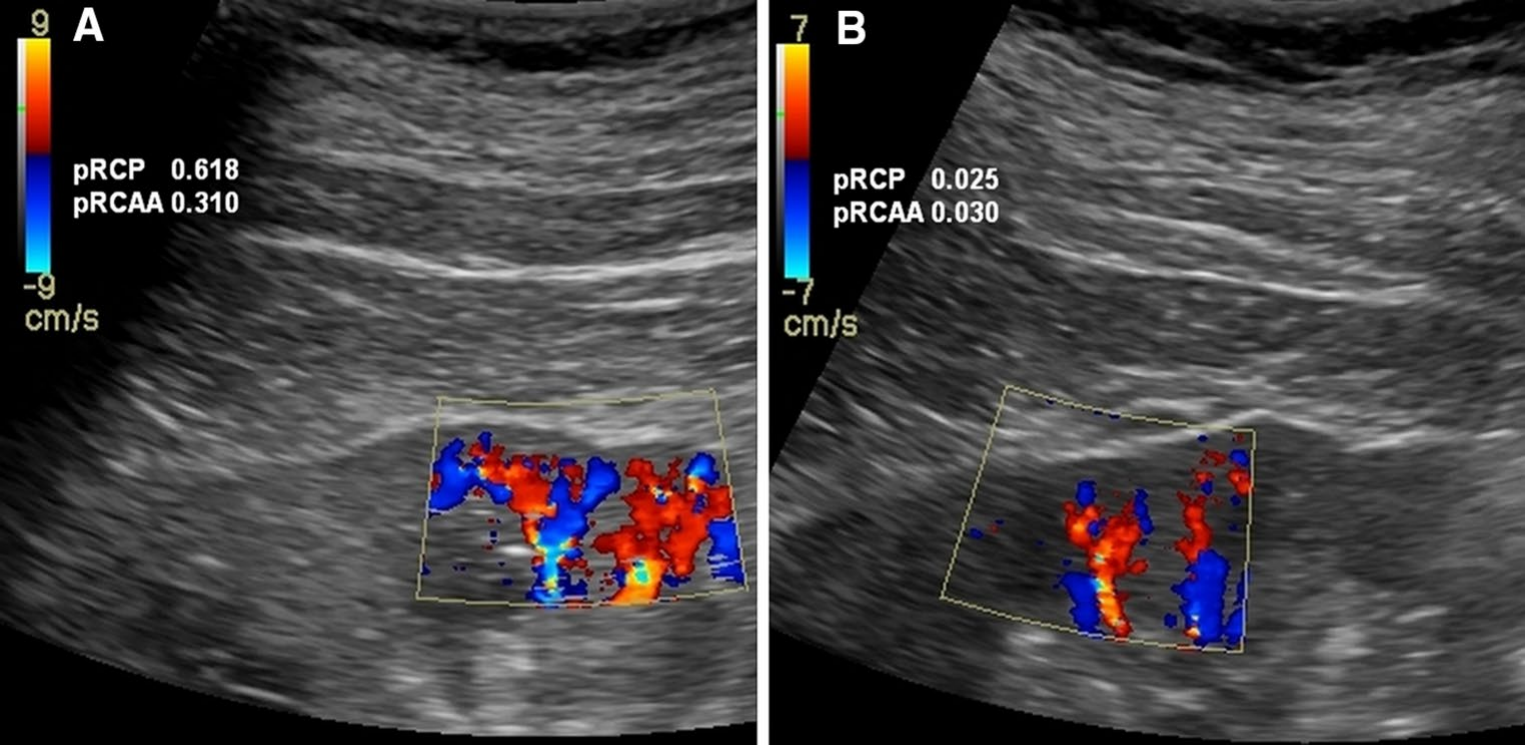
Color Doppler imaging of renal cortical perfusion. Right kidney cortical perfusion imaging: **a** 61-year-old man with GN-CKD; **b** 60-year-old man with HT-CKD

Analysis of US Doppler perfusion parameters revealed significantly lower values of proximal RCP, total and especially proximal RCAA in the hypertension-related nephropathy group (Table 4; Fig. 1a, b). Values of distal RCP and distal RCAA did not differ between groups. Neither total nor proximal RCP and RCAA values were significantly correlated with their ROIs, renal lengths, and cortical thicknesses. Moreover, the perfusion and arterial area parameters were also not related to BMI.

**Table 4:** Differences in Doppler parameters of renal arteries in investigated groups

Data	All (*n* = 56)	HT-CKD (*n* = 38)	HT-CKD matched (*n* = 18)	GN-CKD (*n* = 18)	*p* value HT/GN (HTm/GN)
RI (ratio)	0.680 ± 0.073	0.689 ± 0.078	0.656 ± 0.074	0.659 ± 0.056	0.159 (0.632)
tRCP (mL/s)	0.307 ± 0.242	0.289 ± 0.264	0.240 ± 0.145	0.346 ± 0.183	0.052 (0.411)
pRCP (mL/s)	0.262 ± 0.201	0.243 ± 0.216	0.407 ± 0.245	0.305 ± 0.163	0.029 (0.282)
tRCAA (cm^2^)	0.191 ± 0.119	0.178 ± 0.131	0.155 ± 0.079	0.220 ± 0.085	0.028 (0.099)
pRCAA (cm^2^)	0.151 ± 0.090	0.138 ± 0.095	0.123 ± 0.062	0.181 ± 0.068	0.009 (0.012)
pAAI (ratio)	0.244 ± 0.120	0.216 ± 0.101	0.223 ± 0.103	0.302 ± 0.137	0.011 (0.071)

*GN-CKD* CKD due to glomerulonephritis, *HT-CKD* hypertension-related nephropathy, *HTm* HT-CKD matched, *RI* renal resistance index, *tRCP, pRCP* total, proximal renal cortical perfusion, *tRCAA, pRCAA* total, proximal renal cortical arterial area, *pAAI* proximal arterial area index; * median [range]

To eliminate the difference in age and sample size, the oldest consecutive patients from HT-CKD group were excluded, and then remaining HT-CKD patients (HT-CKD matched group) were compared with GN-CKD patients. The groups did not differ in age, renal function, and cardiac properties, but total and proximal RCAA were significantly lower in HT-CKD matched group (Table 1, 3, 4).

In the whole population proximal RCP was significantly associated (*p* < 0.05) with age, CKD-EPI_Cys-Cr_, NT-proBNP, Troponin-I, E/A, E/E′, LVEF, RI, and cIMT (Table 5), but in multivariable regression analysis adjusted to age (model without RI) only LVEF and cIMT were independently associated with proximal RCP (*R* ^2^ = 0.39; *p* < 0.001).

**Table 5:** Significant correlations between perfusion parameters and other variables (*p* < 0.05)

Data	CKD etiology	Age	CKD-EPI	Troponin-I	NT-proBNP	LVEF	E/A	E/E′	RI	CIMT
pRCP	ns	−0.36	0.487	−0.28	−0.50	0.27	0.30	−0.47	−0.52	−0.42
pRCAA	0.41	−0.35	ns	ns	−0.32	ns	0.27	−0.30	−0.36	−0.37

*cIMT* carotid intima-media thickness, *CKD-EPI* based on cystatin (Cys) and creatinine (Cr) chronic kidney disease epidemiology formula, *E/A* ratio of transmitral early (E) to late (A) ventricular filling velocities, *E/E′* ratio of transmitral early filling velocity to tissue Doppler early diastolic mitral annular velocity, *LVEF* left ventricular ejection fraction, *RI* renal resistance index, *ns* not significant

ROC analysis showed that hypertensive kidney damage was identified with a sensitivity of 67% and a specificity of 68% with a threshold for RCP ≤0.21 mL/s (accuracy 0.679; AUC 0.715, *p* = 0.002). Analysis performed for proximal RCAA showed significant correlations with age, etiology of kidney damage, NT-proBNP, E/A, E/E′, RI, and cIMT. Although, in multivariable regression analysis adjusted to age only cIMT was independently associated with proximal RCAA (*R* ^2^ = 0.25, *p* < 0.001), statistical power of pRCAA and etiology of kidney damage association was high (0.90, for the probability of type I error =0.05). Finally, although ROIs did not differ between groups, arterial vascularity (AAI) was significantly lower in HT-CKD compared to GN-CKD patients (Table 4).

**Fig. 2:**
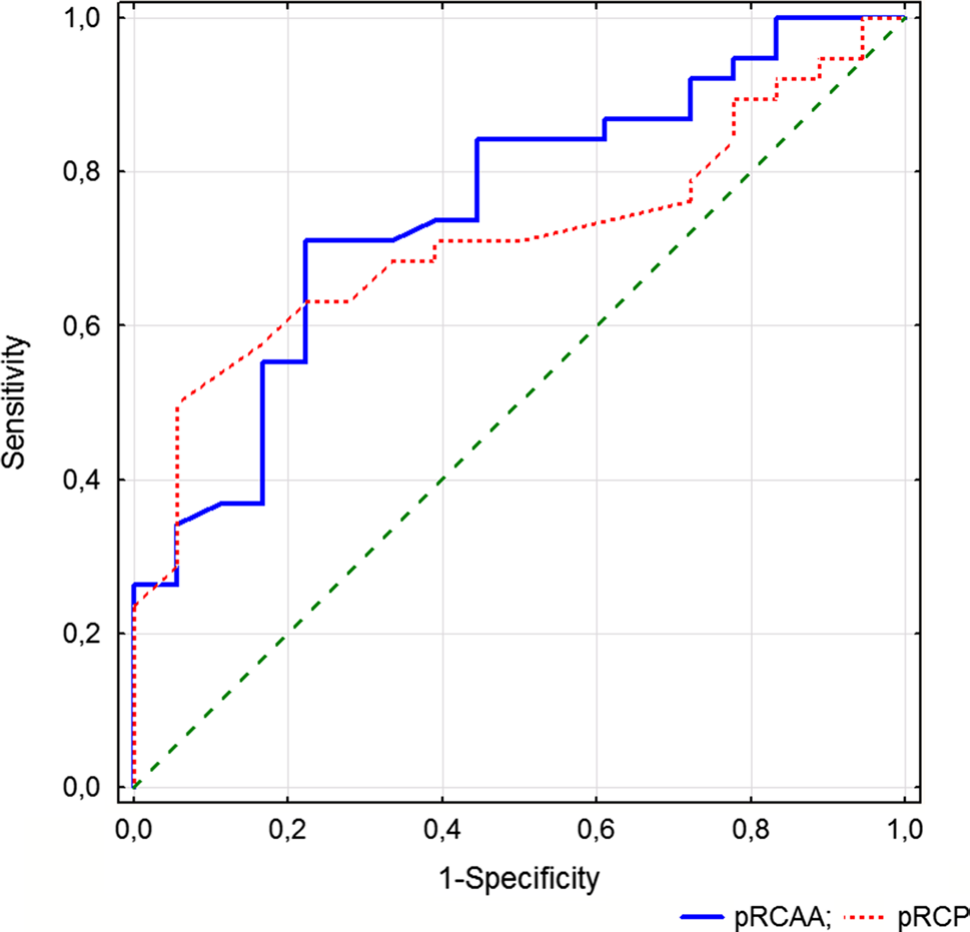
Receiver operating characteristics (ROC) of total proximal renal cortical perfusion (tRCAA, *full line*, threshold ≤0.149 cm^2^) and proximal renal cortical perfusion (pRCP, *dotted line*, threshold ≤0.21 mL/s) to identify hypertension-related kidney damage

Although comparison of proximal RCAA (AUC 0.753, *p* < 0.001) and proximal RCP in ROC analysis did not favor any of these parameters (*p* = 0.422), a threshold of ≤0.149 cm^2^ in proximal renal cortex arterial area (RCAA) identified hypertension-related kidney damage with sensitivity of 71% and specificity of 78% (Fig. 2).

## Discussion

This study shows for the first time that characteristics of US Doppler regional renal cortex perfusion could be helpful in distinguishing between hypertensive and glomerulonephritis-related kidney damage. In ultrasound examinations we found a significant reduction in arterial vascularity in renal proximal cortex in patients with hypertensive kidney damage.

In general it is not difficult to establish the primary diagnosis in advanced stages of cardio-renal disorders [1]. Advanced cardio-renal disturbances are accompanied and preceded by important cardiovascular changes such as hypertension, coronary artery disease, or heart failure. Disorders primarily caused by renal disease are characterized by a decrease in urine specific gravity, changes in urinary sediment (erythrocyturia, leukocyturia), and proteinuria usually exceeding 1.0 g/24 h so that the overall kidney damage exceeds that of the heart. Early diagnosis of the primary cause is the key to start an appropriate treatment and to stop or slow the progress of CKD [20]. However, in the initial stages of the disease, when both the cardiovascular and renal function parameters are within normal limits or only slightly affected, and data from the interview are inconclusive, it is difficult or impossible to identify the underlying cause. Proper identification of the initial etiology of cardio-renal disorders remains an ongoing challenge and is subject of many studies [21, 22]. The correct diagnosis is difficult because of the diversity of causes determining the variants of cardiac (right-/left-sided heart failure, with normal/decreased cardiac output, hypo-/hypertension) or renal dysfunctions (e.g., acute/chronic failure, with/without nephrotic syndrome, glomerular/tubulointerstitial/ischemic damage). Current research and diagnosis of cardio-renal syndrome focuses on identifying appropriate biomarkers such as creatinine, cystatin C, brain natriuretic peptide (BNP), neutrophil gelatinase-associated lipocalin (NGAL), and kidney injury molecule-1 that would allow for an adequate recognition of the primary damage [22, 23]. Recently, Zhou et al. [24] showed an independent correlation of the urea-to-creatinine ratio and left ventricular diastolic dysfunction in 1166 community residents. In a different study, Testani et al. [21] proposed a combined assessment of BNP and blood urea nitrogen-to-creatinine ratio to predict the outcome in patients with decompensated heart failure and renal failure.

Due to the pathophysiology of cardio-renal syndrome and a significant contribution of both organ ischemia and an excess of extracellular volume, the assessment of renal perfusion appears to be a promising direction of research [1]. For example, Ciccone et al. [25] observed the usefulness of US Doppler assessment of renal perfusion in predicting the course of heart failure in 250 patients with heart failure. Schnell and Darmon [26] proposed to assess renal perfusion at the bedside using color Doppler and contrast-enhanced sonography to evaluate RI. We previously reported that RI only slightly correlated with renal perfusion [27]. However, Scholbach et al. [28] proposed an alternative method to quantify renal perfusion which has been termed dynamic tissue perfusion measurement (DTPM). Using this method we previously showed a significant independent association of perfusion intensity (cm/s) in the outer renal cortex with cardiac output, while the only factor independently associated with perfusion intensity in the proximal cortex turned out to be eGFR [5]. Furthermore, we showed that the renal perfusion index (RPI) succeeded in identifying the etiology of chronic cardio-renal syndrome with a sensitivity of 41.7% and a specificity of 83.3% (AUC 0.597) [6].

In the study reported here the groups with different etiologies were comparable in terms of renal function, LVMI, as well as arterial blood pressures. Higher cardiac index in the hypertensive CKD group, despite the lack of differences in LVMI and EF, may have been related to adaptive changes [29]. Another explanation for different CI could be related to myocardial fibrosis as suggested by Edwards et al., who examined 43 patients with primary nephropathies or nephropathies dependent on vasculitis, and who found a significantly greater degree of myocardial fibrosis and significantly lower global longitudinal strain compared with 43 patients with hypertension but without renal failure [30]. This interpretation, however, can be disputed in the present study because of differences in renal function.

In the present study, we showed that arterial alterations expressed as cIMT and cardiac systolic function (LVEF) both had an independent influence on perfusion of renal proximal cortex. An estimated renal function was significantly correlated with this proximal RCP, and as it was previously reported, it was the only independent factor associated with RCP we could find [5]. Recently, Breidthardt et al. [31] published renal perfusion data measured by magnetic resonance imaging in heart failure patients in the presence or absence of cardio-renal syndrome. The authors also found a significant independent correlation of renal perfusion with eGFR confirming the adequacy of DTPM and the plausibility of our observations.

It is evident that organ perfusion should depend on cardiac function and vascular changes. Thickening of cIMT is an established marker of hypertensive target organ damage. Long-term hypertension results in rarefaction of microcirculatory vessels throughout the body, in the myocardium and in renal parenchyma [32]. Comparing the area of arteries we found a significant reduction in HT-CKD compared to GN-CKD patients, mainly in the proximal cortex. The threshold of 0.149 cm^2^ in proximal renal cortex arterial area was a reliable predictor differentiating the etiology of cardio-renal disturbances (sensitivity of 71%, specificity of 78%, AUC 0.753). This observation is new. Previous studies did not analyze the diagnostic potential of arterial area in the proximal renal cortex, obtained by DTPM to distinguish between cardio-renal and reno-cardiac disorders.

In our work, arterial density in the proximal cortex was evaluated as AAI. The significant reduction in arterial vascularity and, at the same time, renal perfusion in HT-CKD patients is probably due to continued pressure-related strain over extended periods of time as documented by higher cIMT, LA, and E/E′ levels in the HT-CKD group [2, 33]. According to current knowledge, the direct arterial damage due to the increased pressure load can be associated with more severe inflammation in the renal cortex [34]. This can explain the differences in perfusion and vessel density in HT-CKD patients, especially because of reduced strain in patients with chronic, stable, and low-activity glomerulonephritis included in this study. Such a possibility is supported by studies of Scholbach et al. [35], who demonstrated a significant reduction in renal cortex perfusion assessed by DTPM with increasing peritubular inflammation.

It is clear that lower and deeply located ROI can easily affect the results of the ultrasound examination. However, in the present study, the assessed perfusion parameters were not significantly correlated with renal length, cortical thickness or ROI size, and BMI, which promotes the protocol and the method that we used. In the study by Tarnoki et al. [36], renal width appeared to be a better marker of the organ disease than renal length, which was rather connected with a genetic predisposition. In our work, the groups did not differ in renal length, but the cortical thickness tended to be even higher in the age-matched HT-CKD patients, which was in opposition to the lower arterial area in this group. However, this observation can be related to a direct arterial damage in the HT-CKD group and an expected more advanced renal interstitial fibrosis, and thus to the cortical diminishing as a consequence of chronic glomerulonephritis in GN-CKD patients. Elevated arterial stiffness, higher peak wave velocity (PWV), and cIMT are well-known markers of hypertensive target organ damage. In comparison with the GN-CKD group, in our study, cIMT was higher in the HT-CKD patients, which suggests more advanced arterial alterations in this group. Unfortunately, this difference appeared to be insignificant between the GN-CKD and the age-matched HT-CKD patients, which, however, can be related to a lower sensitivity of cIMT in the detection of arterial alterations in CKD patients [37]. In these conditions, a measurement of PWV or arterial wall stiffness would be a more appropriate examination showing arterial condition.

Despite promising results our work has several limitations. We have not assessed intra- or interobserver variability of DTPM method, because we used the best quality DICOM movie file for perfusion parameters calculation. Moreover, there are no such data in the available literature. In the only one recently published study by Stoperka et al. [38], the authors suggested limited accuracy of DTPM in diabetic patients. However, in this work, different color gain levels were used to achieve better-quality images, which is strictly restricted to this method, and keeping constant gain has a fundamental importance for the correctness of calculated results [14]. Our study involved only men so that the results cannot be extended to the females. Moreover, incoherent interviews concerning the origin and the precise duration of the disease, as well as the lack of histopathological confirmation could result in inappropriate determination of the causes of the observed differences. Patients with other causes of kidney diseases such as primary tubulointerstitial kidney injury, diabetes, amyloidosis, acute and advanced heart failure were excluded from the study to obtain homogeneous groups. This exclusion limits the usefulness of the test. Another limitation is an exclusion of renal artery stenosis based only on anamnesis and ultrasound Doppler examinations. On the other hand, the included patients did not have clinical indications for computed tomography or magnetic resonance diagnostics. Therefore, and in spite of the promising results of this study, the possibility of using a noninvasive assessment to diagnose the initial cause of cardio-renal disturbances needs to be confirmed in further studies.

## Conclusions

Arterial vascularity and perfusion of renal proximal cortex could be helpful to diagnose the initial cause of cardio-renal disturbances in hypertensive males in early CKD stages. The utility of these measures to diagnose the primary cause of cardio-renal dysfunction in women requires further studies.
